# MRSA outbreak was controlled with daily hexachlorophene showers and hygiene education.

**DOI:** 10.3201/eid1104.041094

**Published:** 2005-04

**Authors:** Dao M. Nguyen, Laurene Mascola, Elizabeth Bancroft

**Affiliations:** * Los Angeles County Department of Health Services, Los Angeles, California, USA;; † Centers for Disease Control and Prevention, Atlanta, Georgia, USA

**Keywords:** disease outbreaks, Methicillin Resistance, Staphylococcus, Football, Hexachlorophene, research

## Abstract

An outbreak of community-associated methicillin-resistant *Staphylococcus aureus* (MRSA) skin and soft tissue infection (SSTI) occurred in a college football team from August to September 2003. Eleven case-players were identified, and boils were the most common sign. Linemen had the highest attack rate (18%). Among 99 (93% of team) players with cultured specimens, 8 (8%) had positive MRSA nasal cultures. All available case-players' MRSA isolates characterized had the community-associated pulsed-field type USA300. A case-control study found that sharing bars of soap and having preexisting cuts or abrasions were associated with infection. A carrier-control study found that having a locker near a teammate with an SSTI, sharing towels, and living on campus were associated with nasal carriage. Successful outbreak control measures included daily hexachlorophene showers and hygiene education.

## Abstract 2

MRSA outbreak was controlled with daily hexachlorophene showers and hygiene education.

Football-related skin infections have gained national notoriety and public interest (1,2). Media coverage of high-profile athletes and teams with skin and soft tissue infection (SSTI) has provided more impetus for research of these infections. Annually, 60,000 college football players compete among 600 teams (3). The community-associated methicillin resistant *Staphylococcus aureus* (CA-MRSA) strains have been a cause of SSTI outbreaks among athletes participating in football, wrestling, rugby, soccer, fencing and canoeing (4–7; Jon Rosenberg, pers. comm.; Los Angeles County Department of Health Services, unpub. data). SSTIs (pustules, "insect bites," boils, and abscesses) are the hallmarks of CA-MRSA infections (8,9). CA-MRSA causes disease in young, otherwise healthy persons without the usual risk factors for MRSA infections (9). In addition, CA-MRSA has unique molecular markers (SCC*mec*IV and Panton-Valentine leukocidin) and fewer resistance genes to non β-lactam antimicrobial drugs than healthcare-associated MRSA strains (10,11).

In August 2002, the Los Angeles County Department of Health Services (LACDHS) received reports of 2 college football players (players X and Y) on team A hospitalized for SSTIs due to MRSA, which was later identified as a community-associated strain (USA300) (12). No other MRSA SSTI was reported on team A until 1 year later. On August 25, 2003, an infectious disease physician notified LACDHS of the hospitalizations of 4 different players on team A with MRSA SSTIs. Despite the lack of background SSTI data on this team, the recurrence of infections prompted an investigation with objectives of identifying players with MRSA SSTIs and nasal carriage, conducting epidemiologic studies, implementing outbreak-control measures, and determining the genotype of the outbreak strain.

Team A was a college football program with 107 players on the roster at the time of the outbreak. The team practiced and played 11 of their 13 games on grass fields. Players began their football season with training camp from August 5 to 18, 2003. In camp, players were sequestered and lived together, in suites of 4 per dormitory, to foster camaraderie among teammates. Rigorous practices were held twice daily in the hot, summer weather.

## Methods

### Case Finding

Case-players were defined as team A members with MRSA culture-confirmed SSTIs or SSTIs presumably caused by the USA300 strain in the outbreak period August 5 to September 5, 2003. Because we suspected that disease exposure occurred during camp, we chose the study period from the start of training camp to ≈2 weeks after the end. Our experience with other SSTI outbreaks found that in most persons lesions develop within 2 weeks postexposure to CA-MRSA. To find case-players, we reviewed the trainer's treatment log to identify players with skin lesions who required medical or surgical interventions. We asked the staff to conduct skin inspections of all players. Players were encouraged to report any skin lesion. In addition, we queried the student health center to determine if these infections were prevalent on campus.

### Nasal Carriage Study

As soon as the current outbreak was recognized, a returning player (player X) was suspected to be the source of infection. Player X had 1 of the 2 cases of CA-MRSA SSTIs discovered in 2002. His locker was directly across from the index case-player, and he was a roommate, during camp, of another case-player. Trainers obtained a nasal culture from player X on August 25. On September 3, trainers obtained cultures from the anterior nares of 99 available team members for a nasal carriage study.

### Laboratory Study

MRSA isolates from case-players and nasal carriers were characterized by using pulsed-field gel electrophoresis (PFGE) with the *Sma*I and *Eag*I restriction enzymes (12,13). PFGE patterns of the isolates were compared with the USA300 strain responsible for other SSTI outbreaks in Los Angeles County (14). This strain was previously determined to contain SCC*mec*IV by the Centers for Disease Control and Prevention (L. Yasuda, pers. comm.). We also characterized a sample of methicillin-susceptible *Staphylococcus aureus* (MSSA) isolates from players' nasal cultures.

### Case-control and Carrier-control Studies

On the basis of anecdotal reports of players sleeping in the locker room on used towels and delaying treatment of cuts and abrasions, we hypothesized that poor hygiene habits and compromised skin integrity might predispose players to infection. We designed a standard questionnaire to collect data on player demographics, living situation, football activities, exposure to persons with skin infections, hygiene practices, histories of skin lesions, and clinical symptoms. Trained health department employees administered the questionnaires in person.

We conducted unmatched case-control and carrier-control studies. Controls were selected by jersey numbers, by using a random-number generator, from asymptomatic teammates without nasal carriage of MRSA. Teammates with positive nasal cultures for MRSA were considered carriers. Carrier-players were defined as carriers with matching PFGE pattern to the USA300 strain. We excluded non-USA300 carriers who might represent the background prevalence of MRSA in the community. Players who were not available for interviews were not included. Bivariate analysis was completed by using Fisher exact test in Epi Info version 3.3 (CDC, Atlanta, GA, USA). Statistical significance was defined as p values <0.05. Because of the small sample size and zero-valued cells, similar risk factors from the bivariate analysis were grouped into categories. Multivariate analysis was completed by using the conditional exact test in SAS version 8 (SAS Institute Inc., Cary, NC, USA).

### Outbreak Control Interventions

Upon recognition of the outbreak on August 25, team A instituted daily hexachlorophene showers for all players, increased the frequency of cleaning the facilities and athletic gear, disinfected the whirlpool tubs, provided more towels, and posted hand-hygiene signs in the locker room. Once nasal culture results were available, team physicians attempted to decolonize carriers with intranasal mupirocin (15). We recommended improving the timeliness of wound care, barring case-players from playing unless wounds were covered, discouraging the sharing of personal items and tubs, prohibiting sleeping in the locker room, and checking laundry procedures. We also disseminated CA-MRSA educational materials to staff and team members (16).

## Results

### Characteristics of Case-players

**Figure 1:**
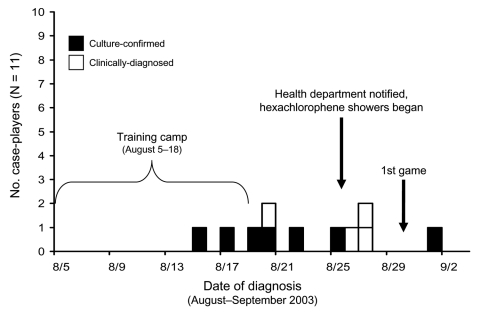
Epidemic curve of clinical and methicillin-resistant *Staphylococcus aureus* skin and soft tissue infections among players on a college football team by date of diagnosis, Los Angeles County, August–September 2003.

**Figure 2:**
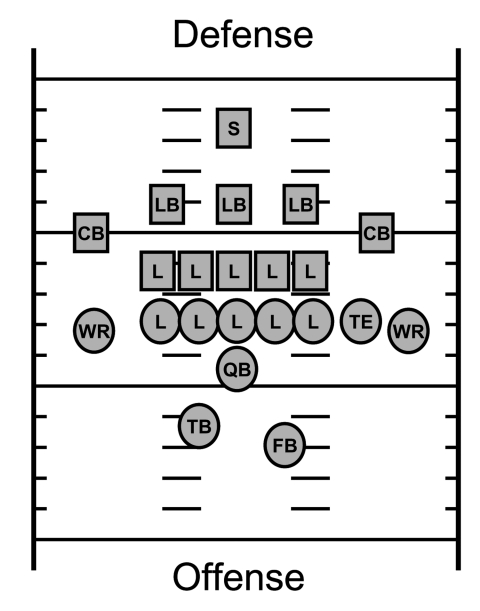
Football field positions; see Table 2 for position-specific attack rates. S, safety; LB, linebacker; CB, cornerback; L, lineman; WR, wide receiver; TE, tight end; QB, quarterback; TB, tailback, FB, fullback.

We identified 11 case-players out of 107 team members for an attack rate of 10%. Cases were diagnosed during or within 2 weeks of the end of training camp (Figure 1). The first case was diagnosed on August 15, the last on September 1. With 1 exception, infections occurred before the first scheduled game on August 30. The most common sign was a boil (Table 1). The elbow was the most common body site infected. No infection was at a current site of skin trauma or occurred at >1 body location simultaneously. Before hospitalization, the index and second case-players were given cephalexin and levofloxacin, respectively, for their infections without any clinical improvement. In total, 4 case-players were hospitalized and treated with parenteral vancomycin. Subsequent nonhospitalized case-players were treated with doxycycline and rifampin. Lesions of 9 players required surgical incision and drainage. All case-players ultimately responded to treatment with resolution of their infections. The median age of case-players was 20 years, with a median tenure of 2 years on team A. Linemen had the highest attack rate (18%) among all field positions (Figure 2, Table 2). No quarterbacks, wide receivers, or special team players (kickers, punters) were affected. All were healthy men without underlying illnesses. Eight (80%) case-players interviewed reported having never worn elbow pads, and 6 (60%) usually did not have cuts or abrasions covered until >1 hour postinjury.

**Table 1:** Characteristics of case-players (N = 11)*

Case-player	Age (y)	Field position	No. y on team	Date of diagnosis	Presenting sign	Site of infection	MRSA+ culture	MRSA genotype†
1	20	Fullback‡	2	8/15/03	Boil§	Knee	Y	A
2	21	Cornerback‡	2	8/17/03	Boil§	Elbow	Y	A
3	20	Linebacker	2	8/19/03	Boil§	Elbow	Y	NA
4	18	Lineman	1	8/20/03	Folliculitis	Leg	Y	NA
5	18	Lineman¶	1	8/20/03	Folliculitis	Knee	NC	–
6	21	Lineman‡	3	8/22/03	Insect bite§	Foot	Y	A
7	18	Lineman‡	1	8/25/03	Boil§	Elbow	Y	A
8	20	Lineman	3	8/26/03	Boil§	Elbow	NC	–
9	19	Lineman	1	8/27/03	Boil§	Forearm	NC	–
10	20	Tight end	2	8/27/03	Insect bite§	Forearm	NC	–
11	20	Cornerback	2	9/1/03	Boil§	Elbow	Y	NA

*MRSA, methicillin-resistant *Staphylococcus aureus*; Y, yes; NA, not available for PFGE analysis; NC, not cultured (clinical diagnosis). †Genotype A is the community-associated MRSA strain (USA300). ‡Hospitalized. §Required incision and drainage. ¶MRSA nasal carrier.

**Table 2:** Position-specific attack rates of clinical and methicillin-resistant Staphylococcus aureus skin and soft tissue infections among players on a college football team

Position (Figure 2)	No. case-players (%), N = 11	No. controls (%), N = 32	Total no. on team (%), N = 107	Position-specific attack rate (%)*
Lineman (L)	6 (55)	11 (34)	33 (31)	18
Tight end (TE)	1 (9)	3 (9)	6 (6)	17
Cornerback (CB), Safety (S)	2 (18)	4 (13)	21 (19)	10
Linebacker (LB)	1 (9)	4 (13)	12 (11)	8
Fullback (FB)/Tailback (TB)	1 (9)	3 (9)	12 (11)	8
Wide receiver (WR)	0	4 (13)	12 (11)	–
Quarterback (QB)	0	2 (6)	6 (6)	–
Special team	0	1 (3)	5 (5)	–

*Attack rate = no. case-players/total no. on team, per position. The overall attack rate = 10%.

### Characteristics of Carriers

**Figure 3:**
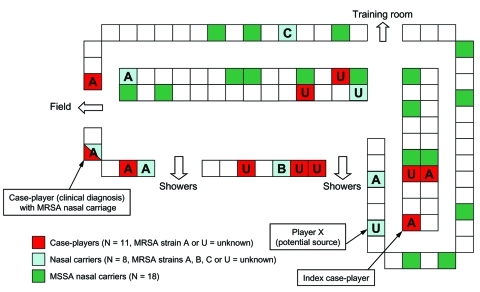
Distribution of locker locations for case-players, methicillin-resistant *Staphylococcus aureus* (MRSA) nasal carriers, and methicillin-susceptible *S. aureus* (MSSA) nasal carriers.

Nasal cultures were obtained from 99 (93%) of 107 team members. Twenty-six (26%) cultures were positive for *Staphylococcus aureus*, among which 8 (8%) were positive for MRSA, including player X. Player Y's nasal culture was negative. The median age of carriers was 20 years (range 18–21 years), and median tenure on the team was 2.5 years (range 1–5 years). MRSA carriage was highest in linemen (38%). We identified 1 case-player with nasal carriage of MRSA. However, trainers obtained nasal cultures after all case-players had begun antimicrobial treatment. Locker room assignments showed clustering of case-players and carrier-players, notably the proximity of the potential source player (player X) to the index case-player (Figure 3). Among MSSA carriers (n = 18), no clustering of locker locations was seen. MSSA carriage was highest among linemen (28%) and cornerbacks/safeties (28%).

### Laboratory Results

Four (57%) of seven MRSA isolates from culture-confirmed case-players were available for PFGE analysis. All were indistinguishable from each other, the USA300 strain found in Los Angeles County, and the isolates from 2 cases (players X and Y) in 2002. We denoted this genotype as strain A. Of 6 (75%) available MRSA isolates from 8 carriers, 4 (67%) were indistinguishable from strain A. Two carriers had unique MRSA genotypes (strains B and C) with ≥7 bands difference between them and between strain A. Strains A, B, and C, player X and Y's isolates, demonstrate community-associated antimicrobial susceptibility phenotypes (Table 3). Among 5 MSSA isolates characterized, all had ≥7 bands difference among themselves as well as from the USA300 strain.

**Table 3:** Comparison of antimicrobial susceptibility patterns for *Staphylococcus aureus* isolates from case-players, carriers, and players X and Y*

Antimicrobial drug	Case-players (2003)	Nasal carriage strains (2003)			Player X		Player Y	
		A†	B	C	Wound (2002)	Nasal (2003)	Wound (2002)	Wound (2003)
Penicillin	R	R	R	R	R	R	R	R
Oxacillin	R	R	R	R	R	R	R	R
Gentamicin	S	S	NT	S	S	NT	S	S
Levofloxacin	I	I	S	R	I	I	I	I
Vancomycin	S	S	S	S	S	S	S	S
Clindamycin	S	S	S	R	S	S	S	S
Tetracycline	S	S	S	S	S	NT	S	S
Rifampin	S	S	S	S	S	NT	S	S
Trimethoprim-sulfamethoxazole	NT	NT	S	S	NT	S	NT	NT

*R, resistant; S, susceptible; I, Intermediate; NT, susceptibility was not tested for that particular antimicrobial drug. †Strain A is indistinguishable on pulsed-field gel electrophoresis from a community-associated MRSA strain (USA300).

### Case-control and Carrier-control Study Results

Ten of 11 case-players were enrolled in the study; 1 was unavailable for interview. During camp, case-players were 15 times more likely than controls to have shared bars of soap with teammates and more likely to have had preexisting cuts or abrasions (Table 4).

**Table 4:** Comparison of selected potential risk factors and characteristics of case-players and carriers, versus controls

Risk factor or characteristic	Case-players (%), N = 10*	Controls (%), N = 32	OR†	95% CI†	p value†
Shared bars of soap with teammates	5 (50)	2 (6)	15.0	1.69–180	**0.005**
Had preexisting cuts or abrasions	10 (100)	20 (63)	Undef	1.08–undef	**0.02**
Shared towels with teammates	2 (20)	1 (3)	7.8	0.34–471	0.14
Had recent "boil"	3 (30)	2 (6)	6.4	0.58–86	0.08
Shared whirlpool tubs with teammates	8 (80)	15 (47)	4.5	0.72–49	0.07
Used whirlpool tubs	8 (80)	16 (50)	4.0	0.63–43	0.09
Had recent "insect bites"	4 (40)	6 (19)	2.9	0.44–17	0.17
Shaved body	4 (40)	12 (38)	1.1	0.19–6	0.59
Used antimicrobial drugs in prior 3 months	1 (10)	6 (19)	0.5	0.01–5	0.46
Chafed skin from athletic equipment	1 (10)	9 (28)	0.3	0.01–3	0.23

	Carriers (%), N = 5‡	Controls (%), N = 32			
Had locker adjacent/across from teammate with skin infection	4 (80)	2 (6)	60.0	3.05–3042	**0.001**
Shared towels with teammates	3 (60)	1 (3)	46.5	2.02–2511	**0.005**
Lived in dormitory, fraternity, or on-campus housing	5 (100)	8 (25)	Undef	2.12–undef	**0.003**
Shared bars of soap with teammates	2 (40)	2 (6)	10.0	0.49–170	0.08
Had recent "insect bites"	3 (60)	6 (19)	6.5	0.57–88	0.08
Slept in locker/training room	5 (100)	19 (59)	Undef	0.52–undef	0.10
Shared whirlpool tubs with teammates	4 (80)	15 (47)	4.5	0.38–236	0.19

*Not including 1 case-player who was unavailable for interview. †Fisher exact test. Values in **boldface** are significant (p <0.05). OR, odds ratio; CI, confidence interval; Undef, undefined or incalculable value. ‡Not including 3 carriers; 1 was unavailable for interview, and 2 had non–strain A genotypes.

Five of 6 carrier-players were available for interviews. Carrier-players were 60 times more likely than controls to have had a locker adjacent to or across from a teammate with an SSTI and 47 times more likely to have shared towels with teammates (Table 4). Carrier-players were more likely than controls to lived on campus in a dormitory or fraternity house. Among carrier-players and controls, players who lived on campus had a higher mean number of roommates than those who lived in off-campus apartments (2.3 vs. 1.5, p = 0.046).

Potential risk factors were grouped into 3 categories: "sharing" (sharing soap/towels with teammates), "skin injury" (cuts, abrasions), and "close contact" (locker adjacent to case-players, living on-campus). Multivariate analysis including these categories indicated that sharing was a significant risk factor for CA-MRSA infection (OR 12.1, 95% CI 1.83–108, p = 0.006) and carriage (OR 17.4, 95% CI 1.03–undefined, p = 0.047).

### Postintervention Surveillance

Daily hexachlorophene showers were in use from August 25 to September 19. No new infections were reported during the 4 weeks after the discontinuation of the hexachlorophene showers. From October 20 to November 9, MRSA SSTI developed in 4 players: a lineman with a chin abscess, a linebacker (player Y from 2002) with an elbow boil, a quarterback (player Z) with folliculitis on a leg, and a tight end with a gluteal boil. Three MRSA isolates (except from the tight end) were available for PFGE; all matched strain A. The lineman in this cluster shared bars of soap with his roommate, a case-player.

Because of ongoing disease transmission and to identify potential reservoirs of MRSA, all 28 staff and student trainers and managers were nasally cultured on November 3; 11 (39%) were positive for MSSA. None was positive for MRSA. On November 22, we observed an official game. Previously unidentified lapses in hygiene practices occurred on the sidelines. We observed that student trainers reused hand towels between players, and players shared towels among themselves. Subsequently, the team switched to single-use towels on the sidelines. No new infections were reported for the remainder of the 2003 season. In the following season (August–December 2004), no MRSA SSTI outbreak occurred on team A. However, player Z had a recurrence of MRSA pustules on the forearm and leg in October 2004. He responded to outpatient treatment with doxycycline, rifampin, and incision and drainage of the lesions. His MRSA isolate was not available for PFGE. Throughout the last 3 football seasons, we received no reports of SSTI outbreaks among opposing athletes after playing this team.

## Discussion

This report is the first of recurring CA-MRSA SSTIs in a football team during consecutive seasons. From 2 cases in 2002 to an outbreak involving 11 players in 2003 and then 1 case in 2004, we have shown that eradicating these infections is difficult once they become established in a football team. Infections were likely propagated year to year from previously infected players, and they appear to be susceptible to recurring colonization and infection themselves.

Consistent with other reports, our findings implicate sharing personal items and improper wound care as risk factors for CA-MRSA infections (17,18). While the concept is counterintuitive, soap sharing was also associated with MRSA infections in a prison outbreak (19). Therefore, teams should consider switching to liquid soaps in an outbreak situation and always provide prompt wound care.

Linemen were identified as a high-risk subgroup. They engage in frequent and aggressive skin-to-skin contact during games, similar to hand-to-hand combat maneuvers as reported in a military MRSA outbreak (20). In addition, linemen tend to be physically larger than their teammates. Increased body mass index and lineman position were risk factors for CA-MRSA infection in another football team outbreak (18).

Two recent reported CA-MRSA outbreaks in football teams detected no nasal carriage in their combined cohort of 182 football players (17,18). In contrast, we document a high MRSA nasal carriage rate (8%) among team A players even while hexachlorophene showers were provided. The actual carriage rate might be higher, since we obtained nasal cultures after all case-players had begun antimicrobial treatment. Additional case-players may have been carriers as well, but they may have been decolonized before culture. Further research is needed to study the association between nasal carriage of CA-MRSA and SSTI to develop decolonization guidelines. The data facilitated a carrier-control study. Similar to risk factors for infection, nasal acquisition of CA-MRSA is associated with sharing personal items, particularly in the locker room.

Crowded living conditions during training camp appear to facilitate the acquisition of CA-MRSA, which then propagates in on-campus housing. Investigators of an outbreak among military recruits found an association between having a roommate with an SSTI and MRSA infection (21). Consequently, players' living arrangements should be as dispersed as possible.

Unique to our investigation are 1 confirmed and 2 presumed community-associated strains of MRSA. We presented laboratory results indicating that the outbreak strain was likely the USA300 genotype. Since we do not have PFGE results from 6 case-players, different strains could have caused those infections. However, a multiclonal outbreak is unlikely, since other MRSA SSTI outbreaks in Los Angeles County among soccer players, men who have sex with men, jail inmates, and newborns have been exclusively due to the USA300 strain (14,22; Los Angeles County Department of Health Services, unpub. data). In contrast, our limited data do not suggest a clonal spread of MSSA on this team. Multilocus sequence typing was not available locally, which prevented further characterization of the isolates.

Selection bias of case-players and controls is a limitation of this study. Enrollment of players with uncultured infections and those without PFGE results introduces the possibility of misdiagnosis and misclassification. Most football teams assign jersey numbers on the basis of field position. Therefore, our control selection method might not have captured a representative sample of the team. However, the distribution of field positions among controls and the entire team appears similar (Table 2). The small sample size produces less precise (wide confidence intervals) results and prohibits more in-depth multivariate analyses. Reporting bias is possible, since players and the team fear negative publicity, and we do not have data on risk factors during the off-season. In order to maintain confidentiality, we were unable to interview several players because of high media scrutiny.

As CA-MRSA strains become more prevalent in the community (23), SSTIs will likely continue to afflict football players. Despite comprehensive infection control interventions, sporadic cases of MRSA SSTIs continue to occur on this team. However, a recurrent outbreak was averted in the latest season likely because of increased vigilance to proper hygiene practices and awareness of this disease among the staff and players.
